# Attentional tuning to color in efficient visual search: Evidence for optimal peak shifts and relational asymmetry

**DOI:** 10.1167/jov.26.8.5

**Published:** 2026-08-21

**Authors:** Dirk Kerzel, Martin Constant

**Affiliations:** 1 Department of Psychology, Université de Genève, Geneva, Switzerland

**Keywords:** visual search, attentional capture, optimal tuning

## Abstract

This study investigated attentional templates and color tuning during efficient visual search. To evaluate optimal tuning and relational accounts, we used a paradigm where participants searched for a color singleton target while ignoring a cross-dimensional shape distractor. Crucially, the distractor's color varied along a precise continuum within the same dimension, spanning values toward and away from the nontarget background. We modeled response times (RTs) and oculomotor capture by fitting asymmetric Gaussian functions to the response curves. Supporting optimal tuning, oculomotor capture (Experiment 1) and manual responses under fixed fixation (Experiment 2) revealed peak interference systematically shifted away from the nontarget color to maximize target-nontarget contrast. Intriguingly, this peak shift was significantly more pronounced in early oculomotor capture than manual RTs, suggesting that optimal tuning biases peripheral attentional guidance (guiding template) more strongly than foveated target verification (target template). Furthermore, response functions exhibited profound asymmetry, displaying shallower slopes for colors extending along the target-defining vector. Whereas Experiment 3 confirmed highly efficient search slopes, Experiment 4 demonstrated that a feature-dissimilar distractor produced negligible capture, validating that selection was governed by a feature-based template. Together, these findings indicate that although attentional templates center on a specifically tuned, contrast-maximizing peak, their overall distribution remains bound to a relative, directional feature rule, consistent with the relational account. Data and scripts are available at https://doi.org/q9f4.

## Introduction

In some search tasks, we need precise information about the target features because the search display contains various features. In other search tasks, the precise target features are less important because they are unique in an otherwise uniform display. Although these two search types are classified as “feature search” and “singleton search” in recent consensus frameworks (Liesefeld et al., 2024), we refer to them here as feature-based and saliency-based search. It is assumed that attention is tuned differently in feature- and saliency-based search. When various features are present in the display, it is necessary to tune attention to the exact target features, resulting in feature-based search. When features in the display are the same except for the target feature, it is sufficient to tune attention to any stimulus that is different from the others, resulting in saliency-based search (Bacon & Egeth, 1994). By saliency, we refer to the local feature contrast of a visual stimulus that allows it to stand out from its immediate surroundings (Nothdurft, 1993). Feature and singleton search differ with respect to their susceptibility to distractor interference. For instance, while a color distractor fails to capture attention when participants search for a specific target shape among heterogeneous nontarget shapes (i.e., feature-based search), it causes substantial interference when searching for a shape singleton among uniform nontarget shapes (i.e., saliency-based search; e.g., Abbasi, Henare, Kadel, & Schubö, 2023; Bacon & Egeth, 1994; Drisdelle, Zivony, & Eimer, 2025; Forschack, Gundlach, Hillyard, & Müller, 2022; Gaspelin, Leonard, & Luck, 2015; Graves & Egeth, 2015; Kerzel & Barras, 2016; Lamy & Egeth, 2003; Lamy, Tsal, & Egeth, 2003; Leber & Egeth, 2006; Ma & Abrams, 2025). This discrepancy arises because attention is guided by a precise feature template during feature-based search, rendering selection immune to salient distractors. In contrast, during saliency-based search, the system is tuned to feature discontinuities in general, leaving it vulnerable to attentional capture by irrelevant singletons (Bacon & Egeth, 1994).

Most evidence distinguishing between feature-based and saliency-based search stems from studies using target and distractor stimuli defined on different perceptual dimensions, such as a shape target paired with a color distractor (Theeuwes, 1991; Theeuwes, 1992). While research on these cross-dimensional distractors shows that interference increases with distractor saliency (Theeuwes, 1991; Zehetleitner, Koch, Goschy, & Müller, 2013), it cannot resolve whether attention is tuned to specific target features. Because the target and distractor belong to separate dimensions, manipulating or even defining their feature similarity is impossible. Had attention been tuned to the target feature, distractors with greater feature similarity should have elicited stronger interference.

Consistent with this idea, visual interference is generally larger when distractors share the target's perceptual dimension (within-dimensional distractors; e.g., Liesefeld, Liesefeld, & Müller, 2022; Liesefeld, Liesefeld, & Müller, 2019). For example, a color distractor captures more attention when the target is also defined by color rather than shape (Gaspar & McDonald, 2014), an effect likely driven by increased target-distractor similarity. Supporting this, when searching for a specific orientation, the interference from orientation-based distractors scales directly with how closely their angles match the target (Donk & van Zoest, 2008; van Zoest & Donk, 2006).

**Figure 1.**
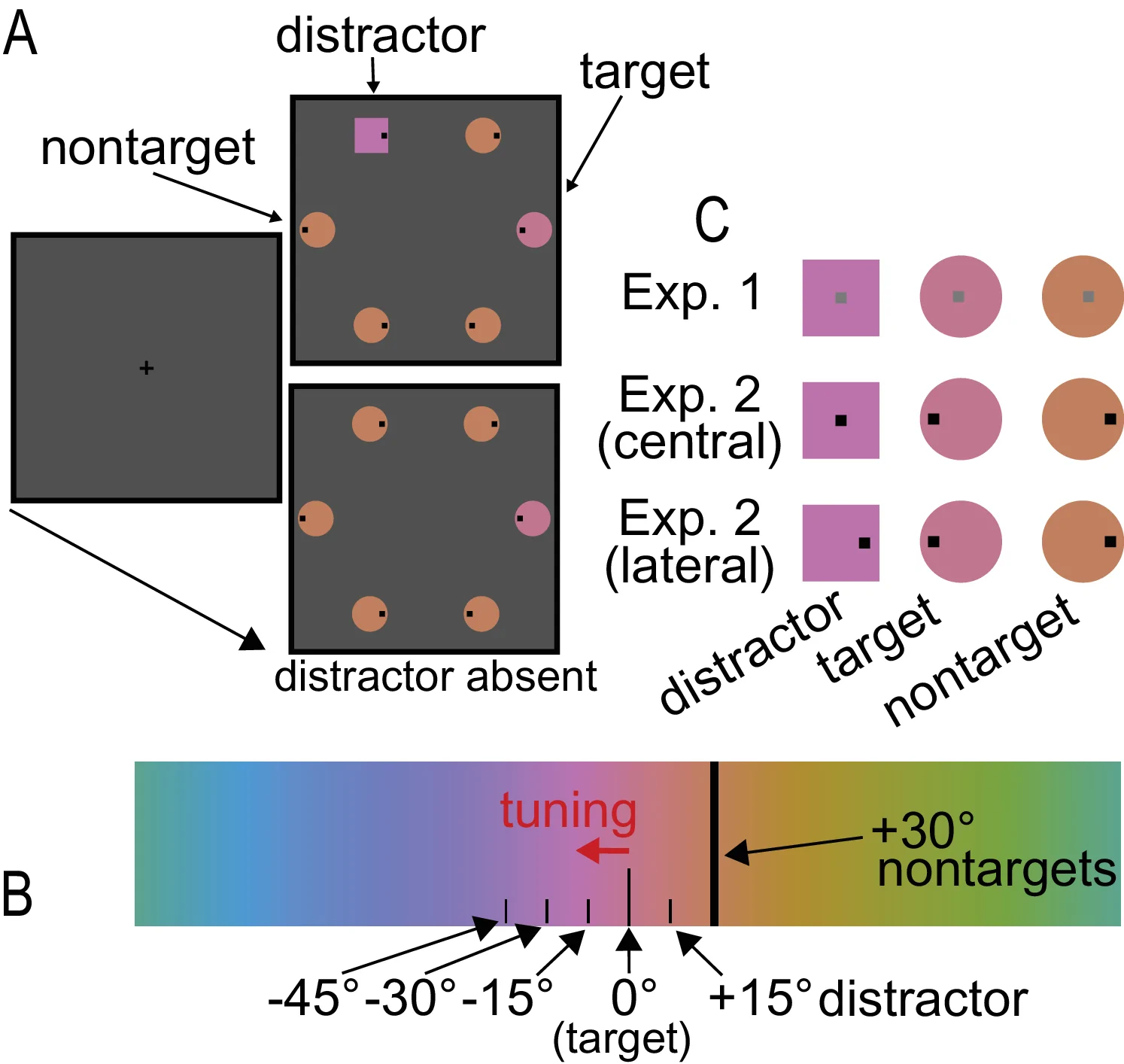
Illustration of experimental stimuli. **(A**) The stimuli and time course. The target was a color singleton, and the distractor was a shape singleton in a variable color. The example shows the distractor in the optimally tuned color at −15°. (**B**) The selection of colors. In a CIELAB-based color space, the distractor colors deviate by −45°, −30°, −15°, 0° or +15° from the target color. (**C**) The dot inside the shapes, which varied between experimental conditions.

Here, we focus on visual search for a chromatic target among colored distractors, a paradigm in which participants likely employ feature-based search to prevent target-distractor confusion. The internal target representation (attentional template; Duncan & Humphreys, 1989; Eimer, 2014) underlying this feature-based search can be modeled by Becker's (2010) relational account. This framework posits that attention is guided not only by specific target features, but also by a relational rule specifying how the target differs from nontargets. For instance, because the target in Figure 1A is *redder* than the nontargets, attention should be guided toward redder stimuli. Supporting this hypothesis, Hamblin-Frohman and Becker (2021) found that oculomotor capture by a within-dimensional color distractor was substantially larger when the distractor was redder than the target (−7°) compared to when it was less red (+7°). Here, “redness” was defined relative to the nontarget color: negative values denoted colors shifting further away from the nontargets (i.e., redder than the target, from −7° to −28°), whereas positive values denoted colors shifting toward the nontargets (i.e., less red, with the target at 0° and the nontarget baseline at +14° in the dissimilar search condition). Crucially, oculomotor capture remained strong and unchanged for distractors spanning −28° to −7° but was virtually absent for those between +7° and +28°. The absence of a peak in this response function indicates that attention was not tuned to a specific color feature, but rather to a feature relation (“redder”).

In contrast to Hamblin-Frohman and Becker (2021), Kerzel (2020) observed a clear, symmetrical peak in the response function, though symmetry was not formally tested. This divergence likely stems from methodological differences; most notably, Kerzel (2020) used distracting cues that preceded the target display, though the cue colors similarly deviated either toward or away from the nontarget color. Crucially, the peak of the response function was shifted −15° away from the nontarget color, aligning precisely with participants' memory for the target, which exhibited an identical −15° shift. This shifted representation, termed “optimal tuning”, is thought to enhance the discriminability of target from nontarget stimuli (Navalpakkam & Itti, 2007; Schulz et al., 2024). A memory bias consistent with optimal tuning has been extensively documented across a variety of paradigms (Chapman, Chunharas, & Störmer, 2023; Hamblin-Frohman & Becker, 2021; Jung, Han, & Min, 2021; Kerzel, 2020; Maith, Schwarz, & Hamker, 2021; Navalpakkam & Itti, 2007; Scolari & Serences, 2009; Yu & Geng, 2019).

## Experiment 1

Experiment 1 aimed to measure attentional interference across a range of distractor colors to evaluate the shape of the underlying response function. According to the relational account, all colors shifted away from the nontarget color are equally potent at capturing gaze (Hamblin-Frohman & Becker, 2021). However, this prediction diverges from the concept of optimal tuning, which posits that attention is tuned to a specific, shifted color value rather than an entire relational category. Specifically, optimal tuning predicts a distinct peak in the response function, resembling the pattern reported by Kerzel (2020).

To evaluate these predictions, we used an oculomotor capture paradigm (Ludwig & Gilchrist, 2002; Theeuwes, Kramer, Hahn, Irwin, & Zelinsky, 1999) in which participants were required to saccade from the fixation cross to a color singleton target. This movement was necessary because the response-relevant details were hard to discriminate in peripheral vision. The color of the nontargets deviated by +30° from the color of the target. Both target and nontargets were disks. On one third of the trials, a distractor square appeared. The distractor square was a shape singleton, which replaced one of the nontarget disks, and was equally likely one of five possible colors (−45°, −30°, −15°, 0°, or +15). We were interested in the response functions relating variations in the distractor color to oculomotor and manual responses. For oculomotor responses, we determined the percentage of trials with first saccades to the distractor, as in a previous study on this topic (Hamblin-Frohman & Becker, 2021). In addition, we also determined the percentage of first saccades to the target. These percentages do not necessarily add up to 100% because first saccades could also go to nontarget stimuli or be invalid. For manual responses, we measured RTs in the main experimental task, which was to report the relative location of a dot inside the target disk. We indexed distractor interference through several dependent variables. For first saccades, more saccades to the distractor and fewer saccades to the target indicate stronger distractor interference. For manual RTs, longer RTs index stronger distractor interference.

These measures allow for fitting psychometric functions to the data. If relational tuning is present, a discrete peak should be absent, at least within the −30° to 0° color range, consistent with Hamblin-Frohman and Becker (2021). In contrast, optimal tuning predicts a distinct peak shifted away from the nontarget color. Because the response functions reported by Hamblin-Frohman and Becker (2021) were shallow for distractor colors shifting away from the nontarget color but highly steep for colors shifting toward it, we fitted asymmetric Gaussian functions to the data. Asymmetric Gaussians accommodate different slopes to the left and right of the peak, allowing us to capture potential skewness in the response profile.

### Method

#### Participants

The number of participants was partially determined by the counterbalancing demands described below. To fulfill these requirements, 24 undergraduate psychology students reporting normal or corrected-to-normal vision participated for class credit (four male, 20 female; age: *M* = 21.1 years, *SD* = 4). In a related study by Kerzel (2020), the sample sizes were between 18–21 and the $\eta_{p}^{2}$ for tuning scores were between 0.428–.592. We mostly performed the critical comparisons with paired- or one-sample *t* tests. G*Power 3.1 (Faul, Erdfelder, Buchner, & Lang, 2009) indicated that we could detect effect sizes as small as *d*_z_ = 0.52 with a sample size of 24 (two-tailed, *α* = 0.050, power = 0.80).

#### Apparatus

The stimuli were displayed on a 22.5-inch LCD monitor (VIEWPixx Lite, 100 Hz, 1920 × 1200 pixels, standard backlight; VPixx Technologies Inc., Saint-Bruno, Canada). Colors were measured with an i1Display Pro (VPixx Edition) colorimeter by X-Rite (Grand Rapids, MI, USA). Head position was stabilized with a chin/forehead rest at a viewing distance of 66 cm. Responses were collected on a RESPONSEPixx Handheld five-button response box (VPixx Technologies Inc., Saint-Bruno, Canada), which had four buttons arranged in a diamond shape and one button in the center. A desktop-mounted EyeLink1000 (SR Research, Ontario, Canada) recorded eye-movements at a sampling rate of 1000 Hz with the standard saccade criteria for cognitive research (i.e., velocity of 30° of visual angle [dva] per second and acceleration of 8000 dva/sec^2^). The Psychtoolbox (Brainard, 1997; Kleiner, Brainard, & Pelli, 2007) and the EyeLink-toolbox (Cornelissen, Peters, & Palmer, 2002) controlled stimulus presentation and response collection.

#### Stimuli

There was a fixation display and a search display (see Figure 1A). The fixation display contained a central black fixation cross (0.2 dva diameter, 0.07 dva linewidth) on an otherwise empty screen. The fixation cross disappeared in the search display to facilitate saccades. The search display contained six colored shapes on a virtual circle with a radius of 8 dva. The stimuli were equally spaced, with two items positioned on the horizontal midline. Target and nontargets were disks (diameter of 2 dva) whereas the distractor was a filled square (side length of 1.8 dva). Inside each disk, there was a small square (side length of 0.2 dva) that was positioned 0.1 dva to the left or right of the center of the disk (see Figure 1C). Inside the distractor square, however, a small square was presented in the center to facilitate distractor rejection. The small squares were gray and isoluminant with the colored disks.

Colors are described in xyY where Y represents the luminance in cd/m^2^. The background was dark gray, xyY = (0.312, 0.332, 24.3). The colors were sampled on an isoluminant color wheel in CIELAB space (see Figure 1B) where distances reflect perceived color differences (Fairchild, 2013). In CIELAB-space, the isoluminant color wheel had a lightness of L* = 59 (corresponding to a luminance of 48.8 cd/m^2^, lighter than the background) and a chroma (saturation) of 64. The xyY coordinates of the colors are available in the OSF repository (Kerzel & Constant, 2025). Each participant was assigned a target- and a nontarget-color which were kept constant throughout the task. There were six possible target colors at rotations of 0°, 45°, 90°, 135°, 180° or 315° on the color wheel. The nontarget colors differed by 30° of rotation from the nontarget color, either clockwise or counterclockwise. We assigned two participants to each of the twelve combinations of nontarget color rotation (30° clockwise, 30° counterclockwise) and target color (0°, 45°, 90°, 135°, 180°, or 315°). Colors were mixed and counterbalanced to control nonlinearities within the color space. Figure 1B shows colors on the color wheel, where color rotation increases from left to right. Color descriptions in the following are always relative. That is, 0° corresponds to the target color and +30° to the nontarget color, regardless of the absolute rotations on the color wheel. On distractor-present trials, the distractor color could be −45°, −30°, −15°, 0°, or +15°. That is, the distractor color could be the same as the target (0°), or further away from the nontarget color than the target (−45°, −30°, −15°) or closer to the nontarget color than the target (+15°). The distractor colors spanned an asymmetrical range around the target color, from −45° to +15°. We selected this distribution to increase sample density around the color values expected to induce maximal interference, thereby capturing the predicted asymmetry around this peak. Specifically, previous research suggests that peak interference occurs for a distractor color shifted away from the nontarget color (Kerzel, 2020). Further, based on Hamblin-Frohman and Becker (2021), we anticipated that interference would decline gradually moving away from the nontarget color, but drop abruptly moving toward it. We address the potential limitations of this asymmetrical distractor distribution in the General Discussion.

#### Design

The distractor was presented on 1/3 of trials and was equally likely to be in each of the five colors. The target and distractor were presented equally often in each of the six positions. There were 540 trials resulting from the combination of two levels of distractor presence (1/3 present, 2/3 absent), five distractor colors (−45°, −30°, −15°, 0°, +15°), six target locations, and three distractor locations. The distractor locations were limited to the three positions opposite the target, which avoided ambiguity as to where the saccade was directed. There were 36 trials for each distractor color, resulting in 180 distractor-present trials, and 360 distractor-absent trials.

#### Procedure

A trial started with the presentation of a central black disk (0.2 dva diameter). When the eyetracker detected that the participant's gaze was within 2 dva of the disk for 300 ms, the disk was replaced by the fixation display, which was presented for a randomly determined duration between 600 to 800 ms. If the eyetracker could not detect the participant's gaze within 2 dva of the disk for more than 5000 ms, recalibration was initiated. After the fixation display, the search display appeared. Participants were instructed to locate the uniquely colored target disk while ignoring the distractor square, and to report the position of the dot inside it using a spatially corresponding button press. That is, they pressed the left button if the dot was on the left side of the disk and the right button if it was on the right. Because the stimuli were isoluminant and the dot's offset was small, a saccade to the target singleton was necessary for discrimination. Participants were instructed to respond as rapidly and accurately as possible. Visual feedback was presented for 1500 ms after trials with choice errors, anticipations, and late responses. We considered trials with RTs shorter than 200 ms as early and trials with RTs longer than 2000 ms as late. Every 135 trials, the eyetracker was calibrated. After calibration, the percentage of correct responses and the median RTs in the preceding trial block were displayed for at least 2000 ms.

#### Analysis and exclusion of first saccades

Saccades were determined by the EyeLink parser and stored in EDF-files, which were converted to a MATLAB-compatible format using the Edf2Mat toolbox developed by Adrian Etter and Marc Biedermann at the University of Zürich (see https://github.com/uzh/edf-converter/). First saccades were not analyzed if their latency was shorter than 80 ms or longer than 300 ms, if their start point deviated by more than 3 dva from the fixation point, if their landing point deviated by more than 4 dva from the nearest stimulus, or if their saccadic amplitude was smaller than 4 dva. Note that the stimuli were on a virtual circle with a radius of 8 dva and separated by 8.4 dva from each other (center-to-center). To determine where the first saccade was directed, we calculated the vector between saccade start and end point. This vector was compared to each of the stimulus vectors between the fixation cross and the stimulus location. The saccade was attributed to the stimulus with the smallest angular difference to the saccade vector.

### Results

#### First saccades

**Figure 2.**
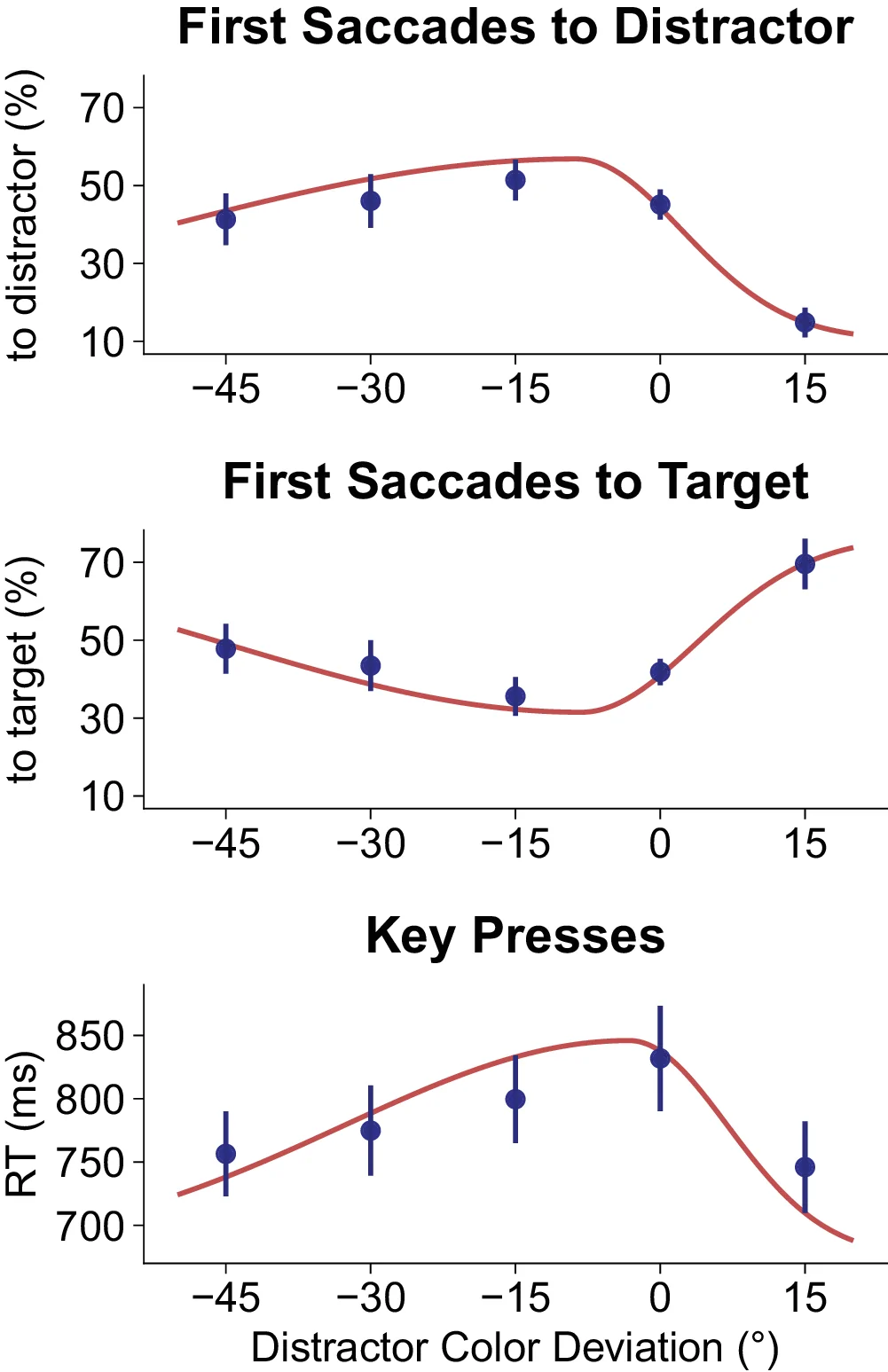
Results from Experiment 1. Upper and middle panels display the percentage of first saccades directed to the distractor and target (distractor-present trials only), respectively, as a function of distractor color deviation. The lower panel displays manual response times in milliseconds. In all panels, the x-axis represents the deviation of the distractor color from the target color in degrees of rotation within the CIELAB space, where 0° denotes the target color and +30° corresponds to the background color. Data points represent the grand means (means of individual participant means), and error bars reflect 95% between-participants confidence intervals calculated using the *t* distribution. Solid red lines depict the asymmetric Gaussian functions generated using the averaged parameters from fits to individual participant data. The fit for target saccades was performed on inverted data (100% - percentage) which was then inverted back for visualization.

Valid first saccades occurred on 78.4% of trials. On these trials, the mean saccadic latency was 167 ms (*SD* = 12) in the distractor-absent condition and 168 ms (*SD* = 11) in the distractor-present condition. To evaluate distractor interference, we calculated the percentage of trials in which the first saccade was directed toward the distractor. As shown in the top panel of Figure 2, the peak percentage of these erroneous saccades was systematically shifted away from the nontarget color. Furthermore, the resulting response functions were highly asymmetrical, exhibiting a steeper decay toward the nontarget color. A similar pattern emerged for target-directed saccades (Figure 2, middle panel). Consistent with the distractor-directed responses, this inverted peak was also shifted away from the nontarget color and the response functions were highly asymmetrical.

#### Manual RTs

Manual responses were classified as erroneous and excluded from analysis if the response time (RT) was shorter than 200 ms, longer than 2000 ms (less than 0.1% of trials), or if a choice error occurred (3.4% of trials). Inspection of choice errors showed no signs of speed-accuracy tradeoff. From the remaining data, we subsequently removed outliers with RTs falling more than 2.5 *SD* above or below the respective condition mean (2.0% of trials). In total, 5.0% of the initial trials were excluded. As illustrated in the bottom panel of Figure 2, the peak of the response function exhibited a slight shift away from the nontarget color, although this displacement was less pronounced than that observed for the proportion of saccades. The characteristic asymmetrical shape of the response function persisted.

#### Model fits

To model the distribution of saccades and manual RTs, for each variable and for each participant, we fitted an asymmetrical (split-width) Gaussian function to their mean responses on distractor-present trials. This function accounts for potential skew in the response profiles by utilizing independent standard deviations for the left and right sides of the peak:

$$\begin{array}{c} f\left(x\right)=A\cdot exp\left(-\frac{{\left(x-\mu \right)}^{2}}{2\sigma^{2}}\right)+C \end{array}$$

with the free parameter σ being either σ_*L*_ for *x* ≤ μ or σ_*R*_ for *x* > μ. The independent width parameters allowed us to capture the systematic asymmetry in the data, characterized by a shallower, less steep onset on the left edge (σ_*L*_) relative to the steeper decay on the right edge (σ_*R*_). *x* is a vector containing the five distractor color offsets (−45°, −30°, −15°, 0°, and +15°). The free parameter μ represents the location (i.e., distractor color) at which the effect of the distractor is maximal, and the free parameter *A* represents the amplitude of this effect. The free parameter *C* determines the baseline level (the height at which the entire function sits). Before fitting, the percentages of first saccades to the target were inverted (100 – percentage) to ensure that the model behaves similarly, but the original values are plotted. Individual fits are presented in Supplementary Figures S1 – S3, whereas bootstrap estimates of individual fit variability are provided in Supplementary Tables S1–S3. During the optimization procedure, parameter bounds were applied to ensure plausible fits. The peak location (μ) was constrained to fall within the physical range of the distractor colors (between −45° and +15°). The width parameter (σ) was constrained to a lower bound of 7.5°; at this minimum, the function decays to 13.5% of its peak amplitude within a 15° interval, matching the spacing between adjacent distractor colors. The upper bound for σ was set to 60°, corresponding to the overall span of tested distractor color values (−45° to +15).

#### Statistical analysis

We focused on two key aspects of the individual functions: the peak location (**μ**) and the slope asymmetry between the left and right sides. To determine if the peak shifted away from the nontarget color (as predicted by optimal attention tuning) we applied one-sample *t* tests comparing **μ** to 0°. We then compared the peak locations between each of the three dependent variables with paired-samples *t* tests. The left-to-right slope asymmetries within each variable were also tested with paired-samples *t* tests. To control for multiple comparisons, we applied the Benjamini-Hochberg False Discovery Rate (FDR) correction separately to each dependent variable (peaks, slopes, and mean RTs; Benjamini & Hochberg, 1995). For transparency, we report uncorrected *p*-values but explicitly note any discrepancies in statistical significance after applying the FDR correction.

#### Data exclusions

For the saccadic distribution analysis, data from two participants were partially excluded: one from the distractor analysis and another from the target analysis. Their estimated individual peaks fell at −45°, indicating they lay outside the range of distractor colors. Conversely, the fits for all response time distributions were satisfactory. Consequently, the final sample size varied slightly across statistical tests (ranging from 22 to 24), maintaining an identical pool of participants with the exception of one or two individuals. No data were excluded for exceeding the upper boundary, as the maximum peak across all dependent variables was +11°.

#### Bayesian statistics

To complement our frequentist analyses, we report the Bayes factor *BF*_10_ for all *t* tests to quantify the strength of evidence that the data provide for the alternative hypothesis (*H*_1_) relative to the null hypothesis (*H*_0_). Following Lee and Wagenmakers (2014)’s conventions, values between 1 and 3 are considered anecdotal evidence, values between 3 and 10 represent moderate evidence, and values greater than 10 indicate strong to extreme evidence. For *BF*s below 1, the corresponding 1/X values support the null hypothesis (e.g., a *BF* between 0.3 and 0.1 indicates moderate evidence for the null hypothesis).

#### Peaks

The peak of the distribution of saccades toward the distractor was significantly shifted away from the nontarget color (−8.7°), *t*(22) = 4.52, *p* < 0.001, Cohen's *d_z_* = 0.94 [0.44, 1.43], *BF*_10_ = 170.24. A similar significant shift emerged for the peak of the flipped distribution of saccades toward the target (−8.2°), *t*(22) = 3.86, *p* < 0.001, *d_z_* = 0.81 [0.33, 1.27], *BF*_10_ = 41.16. For manual response times, the shift was smaller (−3.2°) and only approached significance, *t*(23) = 1.79, *p* = 0.087, *d_z_* = 0.37 [−0.05, 0.78], *BF*_10_ = 0.85.

Comparing the peaks across dependent variables revealed no significant difference between saccades toward the distractor and those toward the target (8.3° vs. 7.3°), *t*(21) = 0.63, *p* = 0.535, *d_z_* = 0.13 [−0.29, 0.55], with moderate evidence for the null hypothesis, *BF*_10_ = 0.27. However, both saccadic peaks showed a greater shift away from the nontarget color than the RT peak. Specifically, the negative shift for distractor-directed saccades was more pronounced than that for RTs (−8.7° vs. −2.8°), *t*(22) = 2.62, *p* = 0.015, *d_z_* = 0.55 [0.10, 0.98], *BF*_10_ = 3.41. Similarly, the peak of the flipped target-directed saccade distribution was shifted further than the RT peak (−8.2° vs. −3.1°), *t*(22) = 2.44, *p* = 0.023, *d_z_* = 0.51 [0.07, 0.94], *BF*_10_ = 2.44.

#### Slopes

For the asymmetrical Gaussian functions used to fit the data, an inverse relationship exists between the standard deviation σ and the slope. Specifically, larger σ values indicate greater data dispersion, resulting in shallower, flatter slopes. Consequently, we predict larger σ values (and thus shallower slopes) on the left side of the distribution than on the right side. Consistent with our prediction, the left slopes were significantly shallower than the right slopes for saccades toward the distractor (44.1 vs. 10.9), *t*(22) = 7.45, *p* < 0.001, *d_z_* = 1.55 [0.93, 2.16], *BF*_10_ = 82709.45. This pattern also held true for saccades toward the target (37.0 vs. 12.0), *t*(22) = 5.71, *p* < 0.001, *d_z_* = 1.19 [0.64, 1.72], *BF*_10_ = 2237.70, and for manual RTs (29.6 vs. 10.2), *t*(23) = 4.53, *p* < 0.001, *d_z_* = 0.92 [0.44, 1.40], *BF*_10_ = 186.37.

#### Comparison of −15° and 0° distractor colors

The fitting procedure suggested that the peak of the distribution was closer to the −15° distractor color for saccadic responses, but closer to the 0° distractor color for manual responses. To test this, we compared the respective proportions of saccades and manual response times using paired-samples *t* tests. The proportion of saccades toward the distractor tended to be higher with the −15° distractor color than with the 0° color (50.6% vs. 44.9%), *t*(23) = 1.77, *p* = 0.089, *d_z_* = 0.36 [−0.05, 0.77], *BF*_10_ = 0.83. This trend points toward greater attentional capture when distractor colors were shifted away from the nontarget color. Similarly, the flipped proportion of saccades toward the target tended to be higher for the −15° than the 0° distractor color (64.3% vs. 58.0%), *t*(23) = 2.10, *p* = 0.047, *d_z_* = 0.43 [0.01, 0.84], *BF*_10_ = 1.38. Note that the *p*-value of 0.047 was not significant after FDR correction for three tests (*p*_FDR_ = 0.071). In contrast, manual RTs exhibited the opposite pattern, revealing significantly shorter latencies for the −15° distractor than the 0° distractor (780 vs. 832 ms), *t*(23) = 2.60, *p* = 0.016, *d_z_* = 0.53 [0.10, 0.95], *BF*_10_ = 3.30. This pattern indicates that manual attentional capture was more pronounced when the distractor color closely resembled the target color.

#### Distractor-absent trials

Although not the focus of the present study, we nonetheless evaluated distractor interference relative to distractor-absent trials. In this analysis, we collapsed across distractor colors. More first saccades went to the target on distractor-absent than -present trials (84.5% vs. 47.3%), *t*(23) = 18.80, *p* < 0.001, *d_z_* = 3.84 [2.66, 5.00], *BF*_10_ = 2.93 × 10^12^, and RTs were shorter on distractor-absent than -present trials (689 vs. 782 ms), *t*(23) = 16.37, *p* < 0.001, *d_z_* = 3.34 [2.30, 4.38], *BF*_10_ = 1.75 × 10^11^. Thus distractor interference was strong with a difference in RTs of 93 ms.

### Discussion

Both oculomotor capture and manual response times demonstrated that interference peaked with distractor colors shifted by −8° to −3° away from the nontarget color. Across observers, the asymmetric Gaussian fits revealed a distinct peak indicating the specific color where oculomotor and attentional capture was maximal. These findings are more consistent with an optimal tuning account than with the relational one, as interference was maximal at a single distractor color rather than remaining uniform across a broader range. However, the asymmetry of the slopes aligns with the relational account, which predicts that colors satisfying a relational rule (e.g., “redder”) will elicit sustained interference, resulting in a shallower slope on that side of the peak.

Furthermore, the peak shift of the response function was smaller (and closer to the exact target color) for manual responses than for oculomotor capture. Notably, both shifts were less pronounced than the −15° shift previously reported by Kerzel (2020), contrasting with the −8° saccadic and −3° manual shifts found here. The most critical divergence between these designs lies in the timing of the distractor presentation. In the current oculomotor capture paradigm, the target, nontargets, and distractor appeared simultaneously, allowing for immediate perceptual comparison among all three elements. In Kerzel (2020), by contrast, the cues preceded the search array, preventing direct comparison and potentially magnifying the impact of internal memory distortions. Consequently, the availability of online perceptual comparisons in the present paradigm may explain why the peak shifts were smaller, and likewise, why distractors sharing similar relations to the nontargets elicited comparable levels of interference.

## Experiment 2

In Experiment 1, because the key press occurred after one or more saccades, manual responses confounded processes related to oculomotor control and the generation of the manual response. Experiment 2 separated these processes by asking participants to maintain fixation. To make the localization task feasible in peripheral vision, the luminance contrast of the dots and the dot separation were increased. Two versions of the experiment were run. In one version, the dot inside the distractor square was in the center (as in Experiment 1), and in another version, it was on the left or right. Comparing the central-dot and lateralized-dot version was necessary because distractor rejection may have been easier with the central-dot version where the distractor varied from the remaining stimuli not only in shape, but also in the dot location.

### Method

The methods were as in Experiment 1 with the following exceptions. The central fixation cross stayed on the screen when the search display was presented. Participants had to maintain fixation on the cross until 500 ms after stimulus onset. This time interval appears sufficiently long because the saccadic latency in Experiment 1 was about 168 ms. The dots were changed to increase their visibility in peripheral vision (see Figure 1C). That is, the luminance of the dot was changed from light gray to black and the distance between the center and the dot was increased from 0.1 to 0.8 dva. Because these changes made the task easier than in Experiment 1, we lowered the time-out criterion to 1,250 ms. In the central-dot version of the experiment, the dot was in the center of the distractor square whereas it was on the left or right in the lateralized-dot version. Twenty-four participants were assigned to each version of the experiment. The data from three participants in the central-dot version were discarded because more than 25% of trials had to be removed due to saccadic or manual errors. The 25% criterion corresponds to the mean percentage of rejected trials in the remaining sample plus three standard deviations (*M* = 10%, *SD* = 5). Thus, the final sample was composed of 21 participants in the central dot version (four male, 17 female; age: *M* = 20.0 years, *SD* = 1.7) and 24 in the lateral dot version (three male, 21 female; age: *M* = 21.8 years, *SD* = 5.1).

### Results

We excluded trials if the key press occurred earlier than 200 ms or later than 1,250 ms (0.4% of trials), if a choice error occurred (3.6% of trials; central dot: 4.0%, lateral dot: 3.2%), or if there was a break of fixation or blink within 500 ms after display onset (4.3% of trials; central dot: 5.1%, lateral dot: 3.6%). Inspection of choice errors showed no signs of speed-accuracy tradeoff. In addition, we excluded trials that were 2.5 *SD* above or below the respective condition mean (2% of remaining trials). In total, 12% of trials were excluded.

To evaluate differences between the two versions of the experiment, we conducted a mixed 2 (version: central dot, lateral dot) × 5 (distractor color: −45°, −30°, −15°, 0°, +15°) ANOVA. Mauchly's test indicated that the assumption of sphericity was violated, so we corrected the degrees of freedom using the Greenhouse–Geisser correction. There was an effect of distractor color, *F*(3.2, 13.4) = 138.44, *p* < 0.001, $\eta_{p}^{2}$ = 0.365, but no effect of version, *F*(1, 43) = 0.04, *p* = 0.846, $\eta_{p}^{2}$ < 0.001, nor interaction, *F*(3.2, 138.4) = 1.59, *p* = 0.192, $\eta_{p}^{2}$ = 0.036. Therefore, we collapsed the data across the two versions.

**Figure 3.**
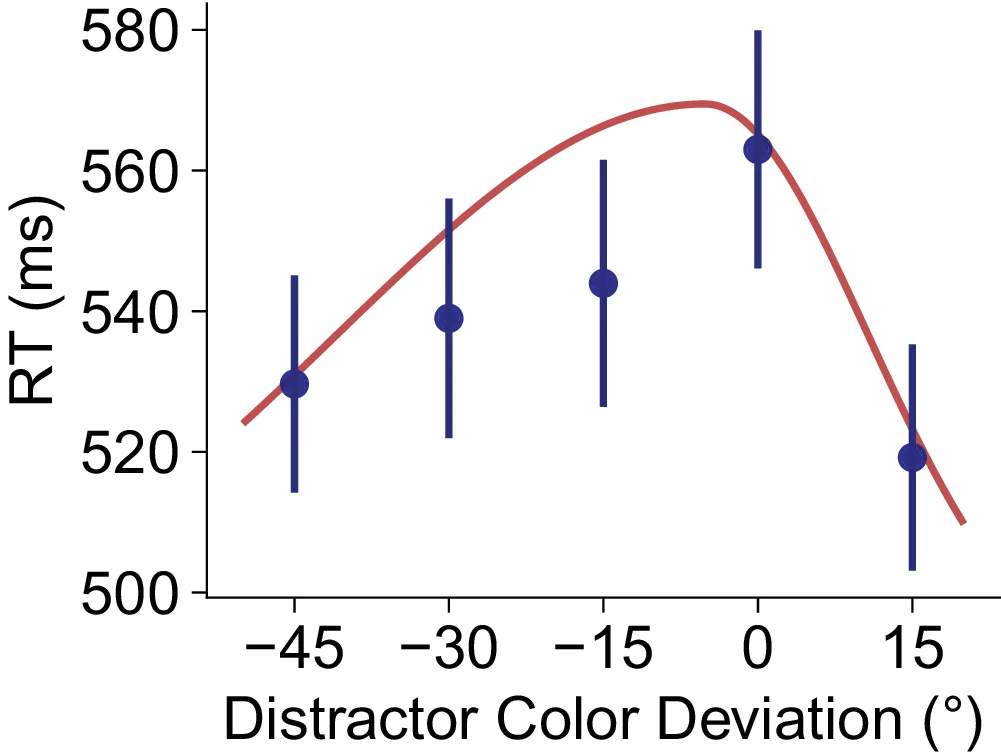
Results from Experiment 2. Manual RTs in milliseconds as a function of distractor color deviation are shown. Data points represent the grand means (means of individual participant means), and error bars reflect 95% between-participants confidence intervals calculated using the *t* distribution. The solid red line depicts the asymmetric Gaussian function generated using the average parameters from fits to individual participant data.

Mean response times and the corresponding model fits are displayed in Figure 3. Individual fits are presented in Supplementary Figure S4, whereas bootstrap estimates of individual fit variability are provided in Supplementary Table S4. The response functions of two participants were essentially flat, characterized by an amplitude parameter smaller than 10; consequently, these datasets were excluded from the analysis of the fit parameters. For the remaining sample, the peak of the distribution of RTs shifted significantly away from the nontarget color (−4.9°), *t*(42) = 2.37, *p* = 0.023. However, the effect size was relatively small, *d_z_* = 0.36 [0.05, 0.67], and the evidence in favor of the alternative hypothesis was only anecdotal, *BF*_10_ = 1.99. Mirroring the pattern observed in Experiment 1, the distribution was significantly shallower on the left side than on the right side (34.6 vs. 15.3), *t*(42) = 4.13, *p* < 0.001, *d_z_* = 0.63 [0.30, 0.95], *BF*_10_ = 146.35.

Furthermore, we analyzed mean RTs across all participants. RTs were significantly longer for distractors in the target color (0°) than for those in the −15° color (563 vs. 544 ms), *t*(44) = 4.17, *p* < 0.001, *d_z_* = 0.62 [0.30, 0.94], *BF*_10_ = 170.92. This finding indicates that attentional capture was more pronounced for target-colored distractors than for distractor colors shifted away from the target color. Finally, RTs on distractor-absent trials were 44 ms shorter than on distractor-present trials (495 vs. 539 ms), *t*(44) = 14.95, *p* < 0.001, *d_z_* = 2.23 [1.68, 2.77], *BF*_10_ = 6.86 × 10^15^.

### Discussion

We excluded contributions of oculomotor processes to manual RTs by asking participants to maintain fixation during 500 ms after stimulus onset. Because the contrast and separation of the dots was changed, the task was feasible without moving the eyes. As a result, RTs dropped from 766 ms in Experiment 1 to 532 ms in Experiment 2. However, the main characteristics of the manual response function from Experiment 1 were unchanged. The manual response function was asymmetrical, and the peak was slightly shifted away from the nontarget color. Thus optimal tuning occurred for both saccadic and manual responses, but the effect was less pronounced in the latter. Furthermore, we ruled out that the location of the dot in the distractor square was important because there were no significant differences between the central- and lateralized-dot versions of the experiment.

## Experiment 3

The findings from Experiments 1 and 2 demonstrated that despite searching for a visually salient color singleton, participants’ attention was specifically tuned (or biased) toward a predefined target color. Highly efficient visual search, such as a singleton search, is conventionally characterized by search slopes near zero, where RTs show very little or no increase as the number of nontargets (set size) increases (Theeuwes, 1991; Treisman & Gelade, 1980). Experiment 3 served as a control to validate that our specific search task met the criteria for efficient singleton search. To achieve this, we manipulated the set size between 4, 6, and 8 items and measured search efficiency by calculating the slope of the linear RT function. Search increases below the 10 ms/item threshold are generally considered indicative of highly efficient search (Wolfe & Horowitz, 2004). A secondary, yet critical, goal of Experiment 3 was to confirm the participants' capacity for distractor rejection based solely on shape. In this specific condition, the color distractor was made identical to the target's color, forcing participants to rely on the target's unique shape for discrimination.

### Method

**Figure 4.**
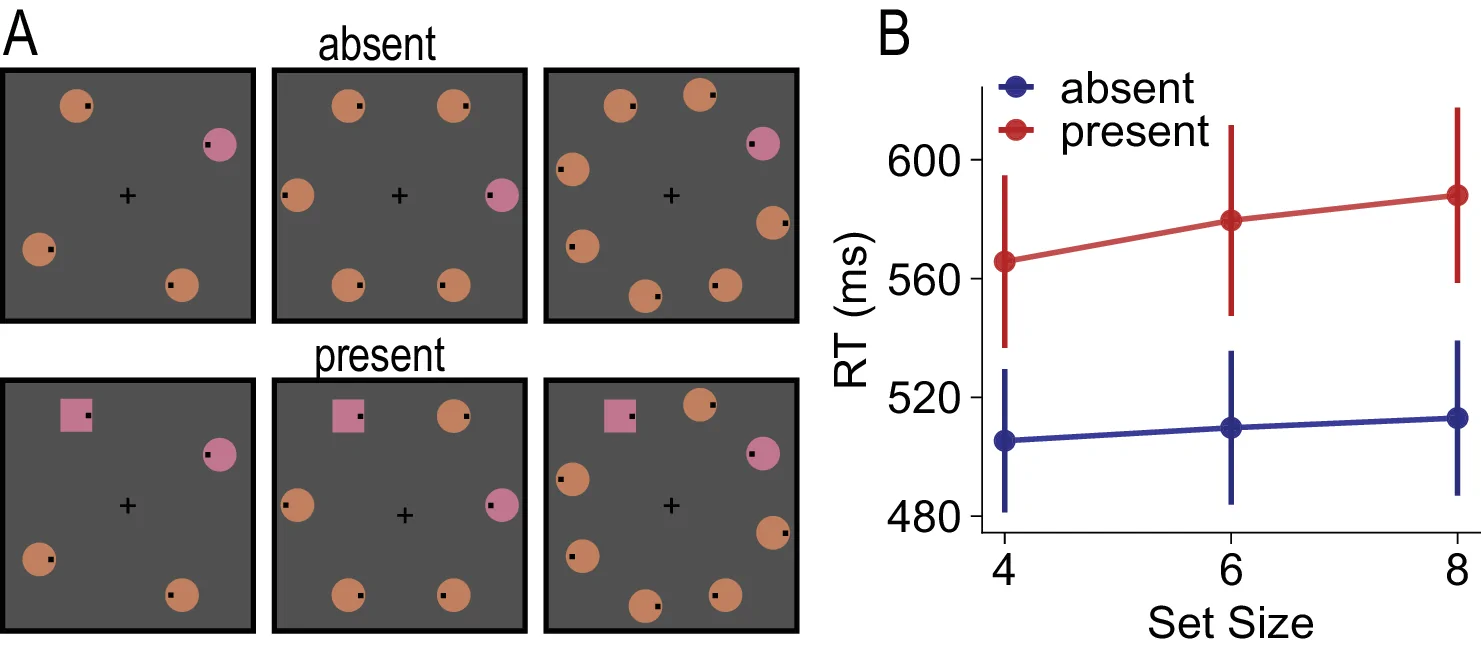
Stimuli and results from Experiment 3. (**A**) The set size manipulation (from left to right) and the absence or presence of the distractor (top vs. bottom). (**B**) Manual RTs as a function of the number of stimuli in the search display (set size) and the presence of a distractor. Error bars reflect 95% between-participants confidence intervals calculated using the *t* distribution.

The procedure for Experiment 3 closely followed the lateral dot version of Experiment 2 with key modifications to the experimental design and stimuli (see Figure 4A). The total number of stimuli (set size) varied randomly on a trial-by-trial basis, with either four, six, or eight items. All stimuli were equally spaced on a circle, and the entire configuration was rotated by a random angle drawn from a uniform distribution (0° to 360°). Random rotations were used to prevent the development of “hot spots” where a stimulus might be presented more frequently. The distractor was always the same color as the target and differed exclusively in its shape, requiring participants to reject the distractor based on the shape feature alone. Consistent with Experiments 1 and 2, the distractor was present in one-third of the trials and absent in two-thirds. Unlike Experiments 1, 2, and 4, here the distractor location was randomly selected from the available nontarget positions; with a set size of four, retaining the previous restriction would have forced the distractor to be invariably opposite the target. Twenty-six participants provided data initially. Following the exclusion of participants with over 25% error trials (based on the threshold from Experiment 2), four participants were removed, leaving a final sample of 22 participants (one male, 21 female; age: *M* = 20.2 years, *SD* = 1.8).

### Results

We excluded trials with RTs shorter than 200 ms or longer than 1250 ms (0.5% of trials), choice error (2.5% of trials), breaks of fixation or blinks (6.2% of trials), and outlier trials (2.4% of remaining trials). In total, 11.0% of trials were excluded. We conducted a 2 (distractor: present, absent) × 3 (set size: 4, 6, 8) repeated-measures ANOVA on mean individual RTs. The group averages are shown in Figure 4B. There was an effect of distractor, *F*(1, 21) = 139.00, *p* < 0.001, $\eta_{p}^{2}$ = 0.869, showing that RTs were 68 ms longer when the distractor was present (578 ms) than when it was absent (509 ms). The effect of set size, *F*(2, 42) = 13.59, *p* < 0.001, $\eta_{p}^{2}$ = 0.393, was modulated by distractor presence in a two-way interaction, *F*(2, 42) = 3.68, *p* = 0.034, $\eta_{p}^{2}$ = 0.149, showing that the increase of RTs with increasing set size was stronger when a distractor was present (566, 580, 588 ms) than when it was absent (505, 510, 513 ms). However, the effect of set size was significant when a distractor was present, *F*(2, 42) = 9.60, *p* < 0.001, $\eta_{p}^{2}$ = 0.314, and when it was absent, *F*(2, 42) = 5.46, *p* = 0.008, $\eta_{p}^{2}$ = 0.206. To quantify the increase in RTs with each additional nontarget, we fit a linear regression to the individual data. The mean regression coefficients were significantly different from zero for both distractor-present and -absent conditions. It was 6 ms/item when the distractor was present, *t*(21) = 3.16, *p* = 0.005, *d_z_* = 0.67 [0.20, 1.13], *BF*_10_ = 9.30, and 2 ms/item when it was absent, *t*(21) = 4.87, *p* < 0.001, *d_z_* = 1.04 [0.51, 1.55], *BF*_10_ = 328.57. Inspection of choice error rates showed no sign of speed-accuracy tradeoff. There were 3.5% choice errors when a distractor was present and 2.1% when it was absent.

### Discussion

Experiment 3 provides evidence that the search task was a highly efficient search. Search slopes were 2 ms/item in the absence of a distractor, increasing only slightly to 6 ms/item when a distractor was present. Both slopes are well below the 10 ms/item threshold, confirming the task's efficiency. Furthermore, the results indicate that participants were able to effectively reject the distractor based solely on its shape. Although the presence of within-dimensional distractors elicited a robust distraction effect (prolonging RTs by 68 ms), this delay did not compromise performance accuracy. The error rate increased by a modest 1%, remaining exceptionally low overall at 3%. This demonstrates participants' ability to reject same-color distractors based on shape alone.

## Experiment 4

To determine whether our findings reflect feature- or saliency-based search, Experiment 4 addresses a core diagnostic ambiguity: while flat search slopes are often attributed to feature-based selection (Bacon & Egeth, 1994; Kerzel & Barras, 2016; Leber & Egeth, 2006), they are equally predicted by saliency-based models (Theeuwes, 1992; Theeuwes, 2004). To untangle these accounts, we introduced a highly salient distractor that was distant from both the target and nontarget colors in color space. If participants rely on a feature-based search template, this distractor should not capture attention because it fails to match the target features. Conversely, if capture occurs, it would demonstrate that selection is driven by physical salience, thereby validating the role of saliency detection in our paradigm.

### Methods

**Figure 5.**
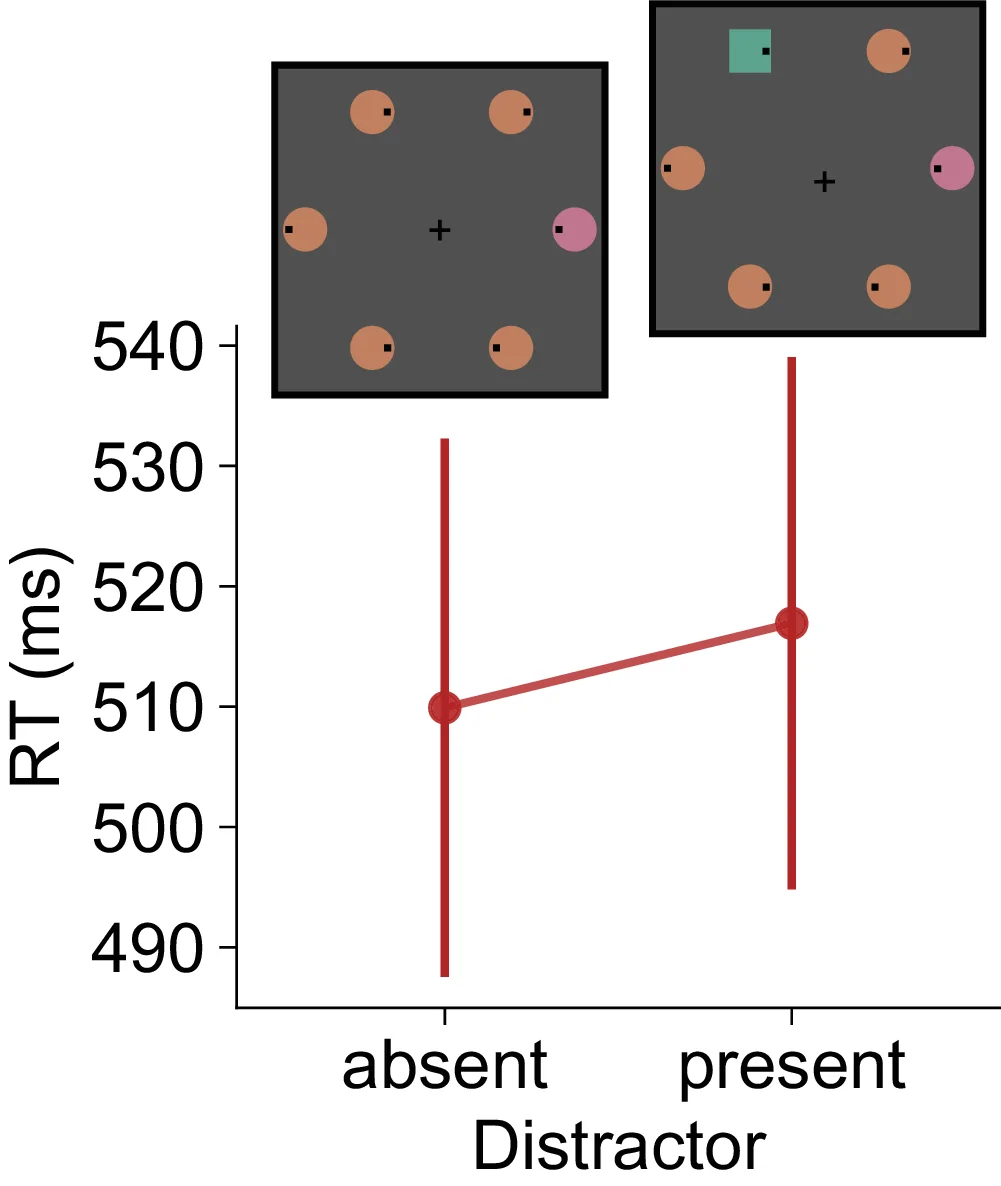
Stimuli and results from Experiment 4. The graph shows manual RTs for distractor-absent and distractor-present conditions. The left inset illustrates a distractor-absent trial, while the right inset illustrates a distractor-present trial where the distractor is distant from the target in color space. Error bars reflect 95% between-participants confidence intervals calculated using the *t* distribution.

As for Experiment 3, the procedure for Experiment 4 was based on the lateral dot version of Experiment 2, with specific modifications to the design and stimuli (see Figure 5). As in Experiments 1–3, the distractor (a square) appeared in one-third of trials and was absent in the remaining two-thirds. To ensure maximum distinctiveness in color space, the distractor's color was always set at a 180° offset from the target's color. Although 25 participants were initially recruited, one was excluded for exceeding a 25% error rate. The final sample consisted of 24 participants (two male, 22 female; age: *M* = 22.6 years, *SD* = 7.4).

### Results

Following the same procedure as in Experiments 2 and 3, we excluded trials with RTs shorter than 200 ms or longer than 1,250 ms (0.5% of trials), choice errors (2% of trials), breaks of fixation or blinks (5.6% of trials), and outlier trials (2.4% of remaining trials). In total, 9.9% of trials were excluded. Group averages are illustrated in Figure 5. The main effect of distractor presence was significant, *t*(23) = 3.83, *p* < 0.001, *d*_z_ = 0.78 [0.32, 1.23], *BF*_10_ = 40.07, with RTs being 7 ms longer in the distractor-present condition (517 ms) than in the distractor-absent condition (510 ms). Analysis of choice error rates revealed no evidence of a speed-accuracy tradeoff; error rates were identical across both the distractor-present (2.0%) and distractor-absent (2.0%) conditions.

### Discussion

Experiment 4 shows that interference from the feature-dissimilar distractor was drastically smaller than from the feature-similar distractor in Experiment 3 (7 vs. 68 ms), suggesting that prior interference was mostly driven by a feature-based search strategy. Although this residual interference was small in milliseconds, the statistical effect sizes remained large ($\eta_{p}^{2}$ of 0.389 and 0.869, respectively). This remaining delay likely reflects non-spatial filtering costs, where highly discriminable distractors slow the allocation of attention to the target without actively capturing attention at their own location (Folk & Remington, 1998; Treisman, Kahneman, & Burkell, 1983).

## General discussion

We investigated attentional tuning to color during efficient, feature-based search. Participants searched for a color singleton target; on 33% of trials, a cross-dimensional shape-singleton distractor appeared, whose color was systematically manipulated to introduce a within-dimensional feature conflict. This manipulation allowed us to evaluate divergent predictions from two prominent accounts of attentional tuning. The relational account (Hamblin-Frohman & Becker, 2021) posits that attention is tuned to a relative feature rule (e.g., “redder than the background”), predicting equivalent oculomotor capture for any color satisfying this relation. Conversely, the optimal tuning account (Navalpakkam & Itti, 2007) proposes that tuning shifts toward a feature value that maximizes discriminability between the target and nontargets. To test these accounts, we fit asymmetric Gaussian functions to the response curves derived from oculomotor capture and RTs across Experiments 1 and 2. Consistent with optimal tuning, the peak of these response functions was systematically shifted away from the nontarget color in the direction that maximizes target-nontarget contrast. However, the functions were highly asymmetric, displaying shallower slopes for colors deviating further away from the nontarget color. This asymmetry is consistent with the relational account, suggesting that relative feature rules sustain high levels of capture for all colors that satisfy the directional relationship.

### Optimal tuning may be stronger for the guiding than for the target template

An unexpected finding was the misalignment between the response function peaks for oculomotor capture and manual responses in Experiment 1. Although the oculomotor peak was reliably shifted away from the nontarget color, the manual shift was attenuated and occasionally non-significant. This divergence supports the view that optimal tuning differentially constrains attentional guidance versus target verification. Early models established that guidance to candidate objects is sequentially followed by identification and decision-making (Neisser, 1963; Sternberg, 1966). Characterizing the representations underlying these stages, Wolfe (2021) distinguished between a “guiding template” that routes attention and a “target template” that confirms target identity.

The performance contrast between our dependent measures in Experiment 1 illustrates this division. Early saccades (168 ms latency) were imprecise and heavily disrupted by distractors (36%–69% correct), whereas manual responses (532 ms latency) were highly accurate (95%–97% correct). This indicates that initial saccades primarily index the guiding template, which utilizes a biased representation to maximize target-nontarget contrast in the periphery. Manual RTs, however, also capture the subsequent target-verification process. Once attention is successfully guided to an item, the target template relies on direct foveal evidence rather than a biased memory template. Consequently, distractors featuring the exact target color generate the greatest manual interference because they differ from the target only in shape. Thus, task demands appear to favor a biased *guiding* template but a less biased *target* template.

To confirm this account, future work must address an important limitation in our current protocol. Because eye movements naturally change the input from peripheral color-driven processing to foveal shape-driven processing, the current design confounds the internal template shift with external sensory changes. Testing this distinction under fixed stimulation conditions represents an essential next step.

### Asymmetric response functions

Furthermore, the response functions across all dependent measures were consistently asymmetric, characterized by a steeper slope toward the nontarget color and a shallower slope away from it. We interpret this asymmetry as partial support for the relational account: the shallow slope observed for colors extending beyond the peak corresponds to values that continue to satisfy the same relative category (e.g., “redder”). However, this asymmetrical pattern could also be accounted for by alternative mechanisms. Yu and Geng (2019) suggested that the attentional template was asymmetrically sharpened when nontargets are linearly separable and similar to the target. These conditions apply to the current displays. Therefore it may be easier to reject distractors deviating toward the nontargets (where the attentional template is sharpened) than away from them.

Alternatively, the response functions might stem from a non-linear relationship between color contrast and saliency. Assuming observers partially relied on saliency detection, interference should increase alongside distractor saliency. Saliency, however, may not increase linearly with color distance from the nontarget color (at +30°). For example, a 15° step from +15° to 0° could alter saliency more drastically than an identical 15° step further away from the nontarget baseline, such as from 0° to −15°. However, the current study cannot decide whether relational coding, asymmetrical sharpening of the attentional template, changes in saliency, or some combination of these mechanisms account for the asymmetrical response functions. Most importantly, we are lacking an independent measure of saliency.

### Caveats

A key limitation of the current study is the biased distribution of distractor colors, which were more frequently “away from” the nontarget color (3/5 of trials) than “toward” it (1/5 of trials). Notably, this design characteristic mirrors the dissimilar-target search condition in Hamblin-Frohman and Becker (2021), which used an asymmetrical layout consisting of four distractor colors away from the nontarget, one toward it (between target and nontarget color), and two situated beyond it (absent in current study). Given that participants are known to rapidly learn and exploit biased features to find targets (Addleman & Störmer, 2023; Golan & Lamy, 2024), this distribution could have potentially increased the tendency to fixate on distractors with colors shifted away from the nontarget. However, this explanation seems unlikely for two reasons. First, previous research using a similar paradigm found that cueing effects remained similarly biased away from the nontarget color, regardless of whether the distribution of cue colors was balanced or not (Kerzel, 2020). This suggests that the biased distribution in our study may not have been the driving factor. Second, the existing literature indicates that repeated or frequent distractor presentations can reduce attentional capture (Gaspelin & Luck, 2018; Müller, Geyer, Zehetleitner, & Krummenacher, 2009; Sayim, Grubert, Herzog, & Krummenacher, 2010; Stilwell, Bahle, & Vecera, 2019; Vatterott & Vecera, 2012). Our findings, however, show the opposite effect, with more frequent “away” distractors leading to increased attentional capture. Furthermore, the fitting procedure encountered no instances where a peak fell beyond the upper parameter boundary (+15°) whereas two peaks fell below the lower bound (−45°) in Experiment 1. Therefore it remains highly unlikely that the asymmetric distribution of distractor colors caused us to miss the true peaks of the response functions. However, the asymmetrical layout provided weak constraints on the distribution's right flank, potentially compromising the reliability of our asymmetry estimates—even though the difference between the flanks remained statistically significant throughout.

## Conclusions

We investigated attentional tuning to color using a cross-dimensional distractor that underwent precise within-dimensional feature variations. Consistent with the optimal tuning account, our response curves revealed a peak shift away from the nontarget color. Intriguingly, the magnitude of this shift diverged across dependent variables, and was more pronounced in oculomotor capture than in manual response times. Furthermore, the response functions exhibited a distinctive asymmetry that aligns with a relational account, though additional underlying mechanisms may also contribute to this pattern.

## Supplementary Material

Supplement 1
