# Supplementary material for: Attentional tuning to color in efficient visual search: Evidence for optimal peak shifts and relational asymmetry
Source: J Vis. 2026 Aug 21;26(8):5. doi: 10.1167/jov.26.8.5 (PMC13505795; doi:10.1167/jov.26.8.5)
Supplement: Supplementary file 1 [file jovi-26-8-5_s001.pdf]

Supplementary Materials for *Attentional tuning to color in efficient visual search: Evidence for optimal peak shifts and relational asymmetry.*

Supplementary Figure 1. *Individual tuning curves and asymmetric Gaussian fits in Experiment 1.*

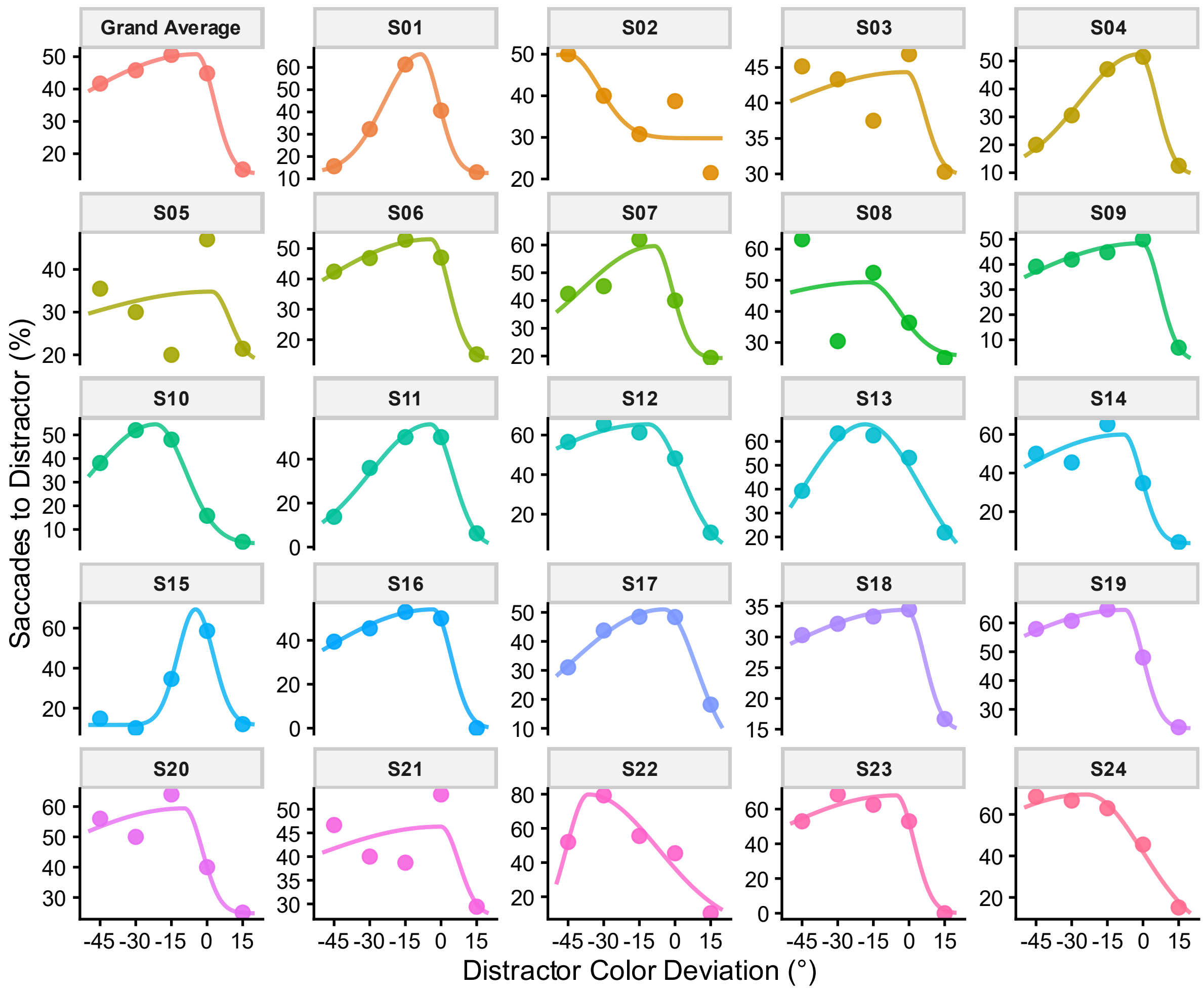

Note. Continuous fitted model curves are overlaid on the corresponding empirical data. Data points represent individual percentages of first saccades directed to the distractor as a function of distractor color deviation. Solid lines depict the individual asymmetric Gaussian functions. The panel labeled "Grand Average" represents the model fit to the grand average data. Parameter estimates from participant "S02" were excluded from subsequent inferential statistics because the estimated peak fell outside the tested empirical bounds (i.e., outside the range of –45° to +15°).

Supplementary Figure 2. *Individual tuning curves and asymmetric Gaussian fits in Experiment 1.*

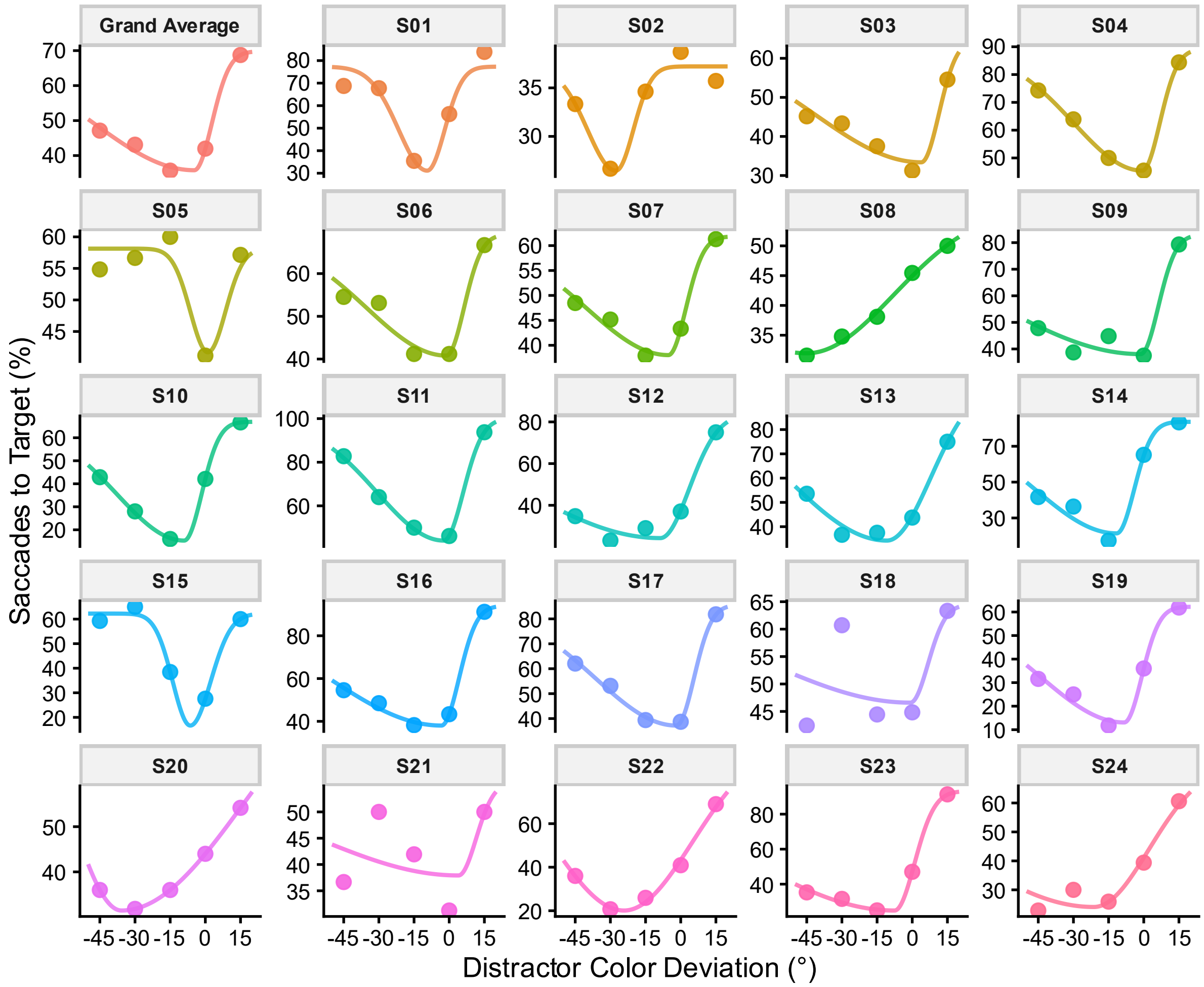

Note. Continuous fitted model curves are overlaid on the corresponding empirical data. Data points represent individual percentages of first saccades directed to the target as a function of distractor color deviation. Solid lines depict the individual asymmetric Gaussian functions. The panel labeled "Grand Average" represents the model fit to the grand average data. Parameter estimates from participant "S08" were excluded from subsequent inferential statistics because the estimated peak fell outside the tested empirical bounds (i.e., outside the range of –45° to +15°). Fits were performed on inverted data (100% – percentage) which were then inverted back for visualization.

Supplementary Figure 3. *Individual tuning curves and asymmetric Gaussian fits in Experiment 1.*

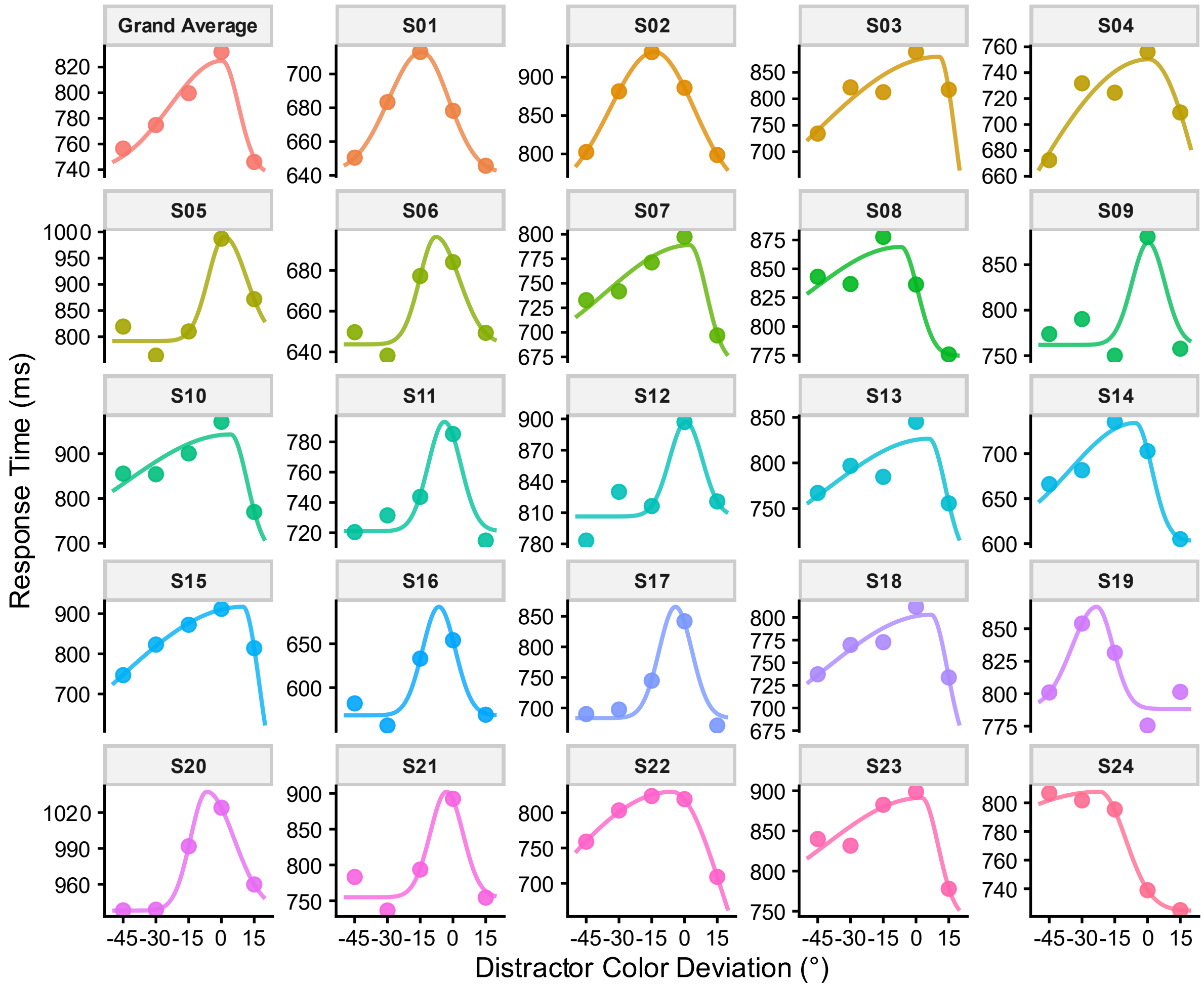

Note. Continuous fitted model curves are overlaid on the corresponding empirical data. Data points represent individual mean response times as a function of distractor color deviation. Solid lines depict the individual asymmetric Gaussian functions. The panel labeled "Grand Average" represents the model fit to the grand average data.

Supplementary Figure 4. *Individual tuning curves and asymmetric Gaussian fits in Experiment 2.*

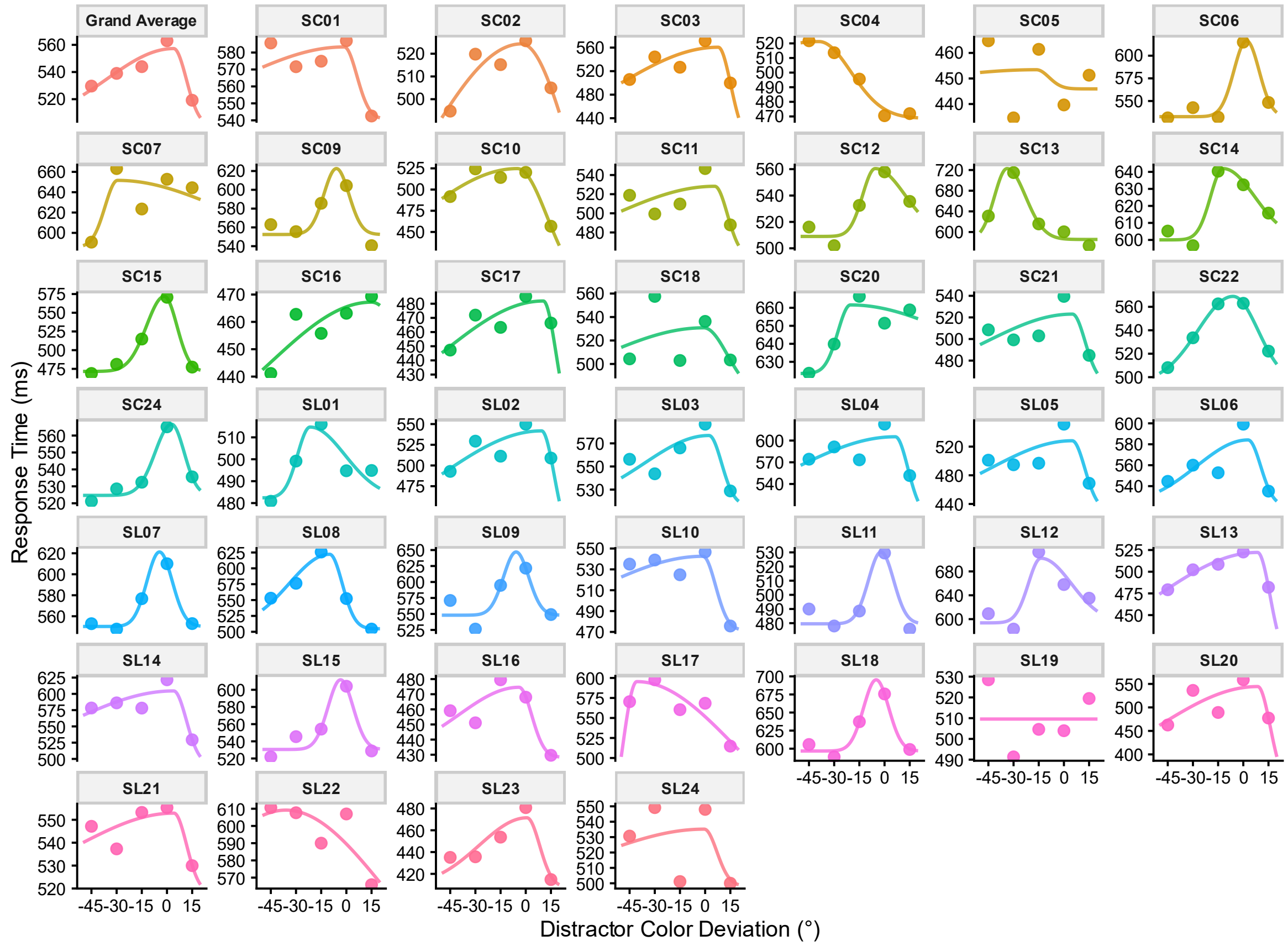

Note. Continuous fitted model curves are overlaid on the corresponding empirical data. Data points represent individual mean response times as a function of distractor color deviation. Solid lines depict the individual asymmetric Gaussian functions. The panel labeled "Grand Average" represents the model fit to the grand average data. Panels label "SC" display participants in the central dot task and those labeled "SL" represent participants in the lateral dot task. Parameter estimates from participant "SC05" and "SL19" were excluded from subsequent inferential statistics due to an essentially flat tuning profile, which precluded a reliable estimation of the peak location.

Supplementary Table 1. *Experiment 1 bootstrap estimates for individual model fits to the percentage of first saccades directed to the distractor.*

ID	*M*(μ)	*SD*(μ)	lo(μ)	up(μ)	*M*(σ_L)	*SD*(σ_L)	lo(σ_L)	up(σ_L)	*M*(σ_R)	*SD*(σ_R)	lo(σ_R)	up(σ_R)
S01	−11.6	5.0	−22.0	−3.4	14.5	6.2	7.5	29.5	11.1	4.7	7.5	21.8
S02	−31.9	16.8	−45.0	1.8	38.1	21.0	7.5	60.0	19.4	17.4	7.5	60.0
S03	−17.4	19.0	−45.0	7.0	34.9	23.7	7.5	60.0	17.7	18.1	7.5	60.0
S04	−3.4	4.8	−13.0	3.9	24.2	11.0	7.5	47.0	8.8	3.2	7.5	16.7
S05	−8.4	16.6	−45.0	6.7	32.9	25.3	7.5	60.0	12.1	13.5	7.5	60.0
S06	−8.8	10.4	−37.9	3.7	43.8	16.1	7.5	60.0	12.0	8.8	7.5	37.0
S07	−11.2	7.0	−30.2	−0.4	32.7	16.6	7.5	60.0	9.9	6.3	7.5	30.9
S08	−23.6	16.8	−45.0	−0.4	50.8	15.5	7.5	60.0	14.7	14.6	7.5	60.0
S09	−5.3	10.3	−45.0	2.5	49.5	13.2	16.3	60.0	10.1	7.7	7.5	38.6
S10	−22.2	8.1	−39.9	−8.9	30.6	17.5	7.5	60.0	12.7	6.0	7.5	27.3
S11	−5.9	5.8	−23.6	1.6	23.1	7.5	8.2	38.0	9.6	3.9	7.5	23.2
S12	−17.9	12.3	−41.1	−1.9	43.0	19.0	7.5	60.0	17.0	9.4	7.5	37.3
S13	−16.4	11.0	−37.2	1.4	27.2	15.1	7.5	60.0	19.3	10.3	7.5	41.8
S14	−11.4	7.3	−34.3	−2.8	45.2	14.5	16.8	60.0	9.8	5.0	7.5	24.8
S15	−4.0	2.7	−8.4	2.1	9.6	5.3	7.5	23.8	8.5	2.4	7.5	14.1
S16	−4.8	5.1	−25.0	−0.9	46.5	12.0	21.4	60.0	8.1	2.9	7.5	18.8
S17	−8.1	11.3	−37.0	5.3	33.2	16.9	7.5	60.0	13.6	10.1	7.5	39.3
S18	−12.9	15.4	−45.0	5.8	35.8	21.7	7.5	60.0	14.8	13.6	7.5	56.4
S19	−17.5	12.8	−45.0	−0.2	41.5	18.6	7.5	60.0	16.0	12.1	7.5	49.8
S20	−16.0	10.2	−45.0	−2.6	42.8	18.2	7.5	60.0	12.2	10.0	7.5	47.0
S21	−11.5	18.2	−45.0	7.0	39.1	23.7	7.5	60.0	15.1	16.9	7.5	60.0
S22	−29.7	10.1	−40.3	−3.7	19.6	16.5	7.5	60.0	23.1	7.5	7.5	34.7
S23	−12.7	11.6	−38.2	−1.6	42.9	17.9	7.5	60.0	12.3	7.1	7.5	27.9
S24	−23.6	12.4	−45.0	−4.4	49.7	16.7	7.5	60.0	20.6	10.4	7.5	42.2

Note. Participant data were bootstrapped (1,000 iterations with replacement) before calculating mean values for each distractor color condition and fitting the statistical model. Reported metrics include the mean, standard deviation, and 95% confidence intervals (lower [lo] and upper [up] bounds) for the $\boldsymbol{\mu}$, $\boldsymbol{\sigma_L}$ and $\boldsymbol{\sigma_R}$ parameters.

Supplementary Table 2. *Experiment 1 bootstrap estimates for individual model fits to the percentage of first saccades directed to the target.*

ID	*M*(μ)	*SD*(μ)	lo(μ)	up(μ)	*M*(σ_L)	*SD*(σ_L)	lo(σ_L)	up(σ_L)	*M*(σ_R)	*SD*(σ_R)	lo(σ_R)	up(σ_R)
S01	−9.1	4.1	−16.9	−0.5	18.2	12.4	7.5	60.0	8.2	3.2	7.5	16.6
S02	−22.8	14.9	−45.0	12.4	22.2	20.3	7.5	60.0	18.5	18.8	7.5	60.0
S03	−5.1	11.5	−40.3	7.5	34.7	22.6	7.5	60.0	13.4	13.5	7.5	60.0
S04	−3.1	5.5	−17.1	4.6	28.3	13.4	7.5	60.0	9.1	4.1	7.5	17.8
S05	−4.9	14.3	−41.8	11.2	22.5	22.0	7.5	60.0	14.1	15.1	7.5	60.0
S06	−6.1	9.4	−33.7	6.0	32.4	20.9	7.5	60.0	12.2	11.3	7.5	58.3
S07	−11.5	12.4	−45.0	6.2	33.7	20.8	7.5	60.0	14.6	13.4	7.5	60.0
S08	−26.5	15.2	−45.0	4.3	33.3	21.6	7.5	60.0	17.4	17	7.5	60.0
S09	−9.5	14.3	−45.0	4.1	48.2	17.2	7.5	60.0	15.0	12.7	7.5	48.0
S10	−14.2	7.3	−29.9	−2.8	28.6	14.8	7.5	60.0	12.5	7.9	7.5	33.9
S11	−4.2	4.7	−20.0	1.9	25.5	8.3	7.5	42.3	8.7	2.9	7.5	16.9
S12	−17.4	13.1	−41.5	−0.4	42.4	19.2	7.5	60.0	19.5	12.0	7.5	46.0
S13	−13.9	12.0	−38.4	3.5	34.7	17.7	7.5	60.0	18.1	11.5	7.5	43.9
S14	−13.8	4.5	−24.1	−7.5	36.2	13.8	15.0	60.0	9.0	3.4	7.5	19.5
S15	−4.8	5.2	−13.1	6.6	13.9	12.6	7.5	58.3	11.5	7.4	7.5	32.1
S16	−4.8	5.2	−21.9	1.7	45.8	12.4	21.6	60.0	8.8	3.6	7.5	19.9
S17	−3.4	5.7	−16.2	4.7	35.6	14.4	7.5	60.0	9.4	4.3	7.5	21.5
S18	−11.1	14.9	−45.0	5.5	40.3	23.6	7.5	60.0	14.9	16.6	7.5	60.0
S19	−12.2	7.2	−28.9	−2.3	36.6	15.9	7.5	60.0	11.6	7.9	7.5	34.6
S20	−23.9	14.0	−45.0	4.2	31.7	21.7	7.5	60.0	19.0	16.7	7.5	60.0
S21	−8.4	15.2	−45.0	7.7	31.5	24.8	7.5	60.0	14.5	16.3	7.5	60.0
S22	−20.3	11.6	−40.7	−1.4	34.4	19.3	7.5	60.0	20.0	11.5	7.5	44.8
S23	−11.9	8.8	−34.8	−2.0	48.1	14.5	15.7	60.0	11.8	6.3	7.5	29.6
S24	−21.0	14.8	−45.0	−1.1	51.5	14.7	7.5	60.0	20.7	17.1	7.5	60.0

Supplementary Table 3. *Experiment 1 bootstrap estimates for individual model fits to the mean RTs.*

ID	*M*(μ)	*SD*(μ)	lo(μ)	up(μ)	*M*(σ_L)	*SD*(σ_L)	lo(σ_L)	up(σ_L)	*M*(σ_R)	*SD*(σ_R)	lo(σ_R)	up(σ_R)
S01	−13.3	8.2	−31.7	2.5	22.1	16.2	7.5	60.0	17.6	15.6	7.5	60.0
S02	−15.3	9.5	−36.4	4.4	23.7	17.8	7.5	60.0	23.0	18.2	7.5	60.0
S03	−3.6	14.3	−29.8	15.0	36.5	23.0	7.5	60.0	31.7	22.0	7.5	60.0
S04	−16.7	16.5	−37.8	12.4	23.6	21.9	7.5	60.0	37.9	22.0	7.5	60.0
S05	1.2	3.9	−5.1	8.6	8.0	2.1	7.5	13.7	14.5	11.9	7.5	60.0
S06	−5.2	9.5	−20.8	15.0	15.0	16.0	7.5	60.0	22.3	18.3	7.5	60.0
S07	−5.5	12.8	−40.5	8.1	39.1	21.9	7.5	60.0	15.6	16.8	7.5	60.0
S08	−13.9	12.5	−45	6.4	32.4	21.8	7.5	60.0	16.3	17.1	7.5	60.0
S09	2.1	3.7	−2.0	8.2	19.7	21.6	7.5	60.0	7.7	2.9	7.5	7.5
S10	2.1	4.4	−5.4	6.8	50.8	15.9	7.5	60.0	7.8	3.5	7.5	7.5
S11	−2.4	7.1	−17.0	9.1	20.9	18.4	7.5	60.0	11.2	9.0	7.5	39.7
S12	0.4	9.5	−25.4	13.1	24.2	20.9	7.5	60.0	15.3	15.5	7.5	60.0
S13	1.4	9.4	−31.0	11.6	29.9	24.1	7.5	60.0	13.0	14.2	7.5	60.0
S14	−7.1	4.5	−20.0	2.5	32.6	11.9	14.4	60.0	11.3	9.6	7.5	45.8
S15	−4.7	12.0	−34.5	10.8	29.3	22.0	7.5	60.0	21.5	18.4	7.5	60.0
S16	−5.6	5.0	−14.1	5.1	12.5	12.0	7.5	60.0	11.3	6.6	7.5	27.1
S17	−1.8	3.7	−7.5	5.9	14.4	10.6	7.5	55.2	8.3	3.0	7.5	15.4
S18	−3.9	13.9	−37.1	15.0	32.6	23.3	7.5	60.0	22.6	20.4	7.5	60.0
S19	−25.6	7.8	−36.1	−5.8	10.6	9.0	7.5	47.6	22.8	21.6	7.5	60.0
S20	−0.9	8.5	−17.9	15.0	18.6	16.7	7.5	60.0	21.4	18.1	7.5	60.0
S21	−0.8	4.1	−6.2	5.7	22.6	23.4	7.5	60.0	7.9	3.8	7.5	10.7
S22	−4.0	9.3	−26.9	7.8	48.0	16.1	7.5	60.0	19.7	16.1	7.5	60.0
S23	−0.5	4.9	−9.4	5.7	43.4	18.5	7.5	60.0	8.0	3.7	7.5	10.3
S24	−21.2	10.9	−45.0	−4.4	36.2	19.2	7.5	60.0	15.1	14.9	7.5	60.0

Supplementary Table 4. *Experiment 2 bootstrap estimates for individual model fits to the mean RTs.*

ID	*M*(μ)	*SD*(μ)	lo(μ)	up(μ)	*M*(σL)	*SD*(σL)	lo(σL)	up(σL)	*M*(σR)	*SD*(σR)	lo(σR)	up(σR)
SC01	−6.6	16.4	−45.0	15.0	32.5	24.2	7.5	60.0	16.0	17.5	7.5	60.0
SC02	−0.7	15.7	−36.0	15.0	29.9	24.0	7.5	60.0	26.8	22.3	7.5	60.0
SC03	3.1	6.6	−8.9	15.0	20.0	19.0	7.5	60.0	12.5	12.0	7.5	60.0
SC04	−33.5	8.7	−45.0	−17.7	27.3	19.7	7.5	60.0	14.0	11.0	7.5	54.8
SC05	−14.8	15.8	−45.0	15.0	40.7	22.9	7.5	60.0	15.7	16.1	7.5	60.0
SC06	0.8	8.3	−35.5	9.5	17.5	20.2	7.5	60.0	9.5	9.3	7.5	60.0
SC07	−7.6	19.2	−39.2	15.0	28.1	24.9	7.5	60.0	24.1	22.9	7.5	60.0
SC08	−23.3	19.2	−45.0	15.0	26.8	23.2	7.5	60.0	20.3	20.4	7.5	60.0
SC09	−1.9	8.2	−38.0	6.0	37.1	23.5	7.5	60.0	9.0	8.2	7.5	30.1
SC10	−22.5	17.9	−45.0	3.0	45.7	21.0	7.5	60.0	36.4	25.2	7.5	60.0
SC11	−2.7	9.7	−45.0	5.7	38.6	24.4	7.5	60.0	9.6	9.6	7.5	60.0
SC12	0.1	9.4	−29.8	15.0	17.5	18.1	7.5	60.0	15.0	15.4	7.5	60.0
SC13	−32.5	8.5	−45.0	−12.6	19.4	17.4	7.5	60.0	16.7	14.8	7.5	60.0
SC14	−5.3	12.1	−36.1	15.0	21.8	20.3	7.5	60.0	22.5	19.3	7.5	60.0
SC15	−1.0	4.2	−7.1	8.0	17.7	16.4	7.5	60.0	8.1	3.0	7.5	13.7
SC16	−9.7	17.2	−36.2	15.0	20.9	20.6	7.5	60.0	33.7	22.3	7.5	60.0
SC17	−0.8	13.9	−37.7	15.0	33.4	24.8	7.5	60.0	19.4	20.0	7.5	60.0
SC18	−19.9	19.6	−41.0	15.0	23.1	23.5	7.5	60.0	27.5	24.5	7.5	60.0
SC19	−24.1	16.8	−45.0	15.0	25.0	20.5	7.5	60.0	23.9	21.5	7.5	60.0
SC20	−12.0	11.7	−30.0	15.0	14.2	14.7	7.5	60.0	27.2	19.5	7.5	60.0
SC21	−2.7	12.2	−43.9	9.0	33.5	24.1	7.5	60.0	12.9	14.3	7.5	60.0
SC22	−10.5	10.5	−29.9	9.4	22.9	19.4	7.5	60.0	23.7	16.7	7.5	60.0
SC23	−8.4	15.1	−44.9	10.8	32.1	23.4	7.5	60.0	18.9	18.9	7.5	60.0
SC24	−1.5	7.6	−18.0	10.6	19.1	18.2	7.5	60.0	13.9	12.1	7.5	60.0
SL01	−21.5	12.1	−37.9	8.0	16.6	16.8	7.5	60.0	32.0	21.0	7.5	60.0
SL02	−1.9	16.2	−38.4	15.0	32.2	25.7	7.5	60.0	18.5	20.1	7.5	60.0
SL03	−1.9	10.6	−45.0	6.4	44.1	22.3	7.5	60.0	10.7	11.8	7.5	60.0
SL04	−0.8	12.7	−40.0	8.3	49.3	19.6	7.5	60.0	13.4	15.8	7.5	60.0
SL05	−1.8	12.4	−45.0	6.4	47.7	20.5	7.5	60.0	10.3	11.4	7.5	60.0
SL06	−4.2	10.0	−45.0	7.3	22.0	21.6	7.5	60.0	11.5	11.1	7.5	60.0
SL07	−5.5	6.6	−18.1	7.8	18.0	16.9	7.5	60.0	11.6	9.1	7.5	41.7
SL08	−12.6	8.7	−39.7	−2.0	39.3	16.5	7.5	60.0	13.7	14.0	7.5	60.0
SL09	−5.0	6.2	−13.3	5.6	22.8	21.6	7.5	60.0	9.2	6.9	7.5	27.0
SL10	−26.3	18.4	−45.0	1.1	45.0	20.6	7.5	60.0	35.1	25.0	7.5	60.0
SL11	−1.5	7.4	−15.2	7.1	22.1	22.7	7.5	60.0	10.2	8.9	7.5	45.1
SL12	−11.9	4.3	−14.9	−6.5	7.8	2.3	7.5	8.0	28.1	17.8	7.5	60.0
SL13	−6.3	13.6	−36.2	15.0	32.4	22.5	7.5	60.0	26.5	20.9	7.5	60.0
SL14	−10.1	17.8	−45.0	7.0	46.4	20.8	7.5	60.0	21.5	21.9	7.5	60.0
SL15	−4.2	14.9	−38.4	10.2	37.5	23.6	7.5	60.0	20.3	19.8	7.5	60.0

SL16	−5.3	7.4	−23.6	5.7	35.6	19.9	7.5	60.0	10.1	9.0	7.5	43.6
SL17	−35.4	13.0	−45.0	−0.5	31.9	22.6	7.5	60.0	35.2	23.3	7.5	60.0
SL18	−4.7	5.2	−12.5	5.9	15.5	16.4	7.5	60.0	9.4	5.7	7.5	20.3
SL19	−24.1	23.7	−45.0	15.0	38.4	20.2	7.5	60.0	23.8	21.2	7.5	60.0
SL20	−1.1	15.8	−38.7	11.7	39.8	25.1	7.5	60.0	17.9	19.9	7.5	60.0
SL21	−5.0	11.1	−37.4	9.0	28.6	22.7	7.5	60.0	14.9	15.3	7.5	60.0
SL22	−17.7	19.2	−45.0	6.7	41.3	21.7	7.5	60.0	19.0	20.1	7.5	60.0
SL23	−0.9	7.7	−11.6	6.8	35.4	24.1	7.5	60.0	8.7	7.4	7.5	18.1
SL24	−14.3	20.9	−45.0	15.0	35.2	25.5	7.5	60.0	18.8	21.3	7.5	60.0
